# 2-[(*E*)-4-(Dimethyl­amino)­styr­yl]-1-methyl­pyridinium 4-chloro­benzene­sulfonate monohydrate^1^

**DOI:** 10.1107/S1600536810043230

**Published:** 2010-10-31

**Authors:** Hoong-Kun Fun, Kullapa Chanawanno, Suchada Chantrapromma

**Affiliations:** a X-ray Crystallography Unit, School of Physics, Universiti Sains Malaysia, 11800 USM, Penang, Malaysia; b Crystal Materials Research Unit, Department of Chemistry, Faculty of Science, Prince of Songkla University, Hat-Yai, Songkhla 90112, Thailand

## Abstract

In the title hydrated mol­ecular salt, C_16_H_19_N_2_^+^·C_6_H_4_ClO_3_S^−^·H_2_O, the 2-[4-(dimethyl­amino)­styr­yl]-1-methyl­pyridinium cation exists in an *E* configuration with respect to the C=C bond and is slightly twisted, with the dihedral angle between the pyridinium and benzene rings being 9.33 (10)°. In the crystal structure, the packing is stabilized by O—H⋯O hydrogen bonds and weak C—H⋯O inter­actions, which link the cations, anions and water mol­ecules into chains propagating in [010]. These chains are stacked along the *a* axis by π–π inter­actions, with centroid-to-centroid distances of 3.6429 (12) and 3.6879 (12) Å; weak C—H⋯π inter­actions are also observed.

## Related literature

For representative bond lengths, see Allen *et al.* (1987 ▶). For background to and application of quarternary ammonium compounds, see: Armitage *et al.* (1929 ▶); Browning *et al.* (1922 ▶); Chanawanno *et al.* (2010 ▶); Wainwright & Kristiansen (2003 ▶); Wainwright (2008 ▶). For a related structure, see: Chantra­promma *et al.* (2010 ▶). For the stability of the temperature controller used in the data collection, see Cosier & Glazer (1986 ▶).
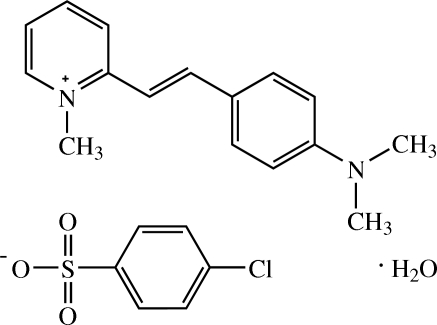


## Experimental

### 

#### Crystal data

C_16_H_19_N_2_^+^·C_6_H_4_ClO_3_S^−^·H_2_O
*M* *_r_* = 448.96
Triclinic, 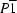

*a* = 6.3895 (1) Å
*b* = 9.8739 (2) Å
*c* = 17.0074 (3) Å
α = 95.721 (1)°
β = 90.500 (1)°
γ = 91.260 (1)°
*V* = 1067.32 (3) Å^3^
*Z* = 2
Mo *K*α radiation
μ = 0.31 mm^−1^
*T* = 100 K
0.31 × 0.10 × 0.05 mm

#### Data collection

Bruker APEX DUO CCD diffractometer
Absorption correction: multi-scan (*SADABS*; Bruker, 2009 ▶) *T*_min_ = 0.912, *T*_max_ = 0.984
23113 measured reflections
6156 independent reflections
4327 reflections with *I* > 2σ(*I*)
*R*_int_ = 0.065

#### Refinement

*R*[*F* ^2^ > 2σ(*F* ^2^)] = 0.057
*wR*(*F* ^2^) = 0.135
*S* = 1.03
6156 reflections
282 parameters
H atoms treated by a mixture of independent and constrained refinement
Δρ_max_ = 0.47 e Å^−3^
Δρ_min_ = −0.43 e Å^−3^

### 

Data collection: *APEX2* (Bruker, 2009 ▶); cell refinement: *SAINT* (Bruker, 2009 ▶); data reduction: *SAINT*; program(s) used to solve structure: *SHELXTL* (Sheldrick, 2008 ▶); program(s) used to refine structure: *SHELXTL*; molecular graphics: *SHELXTL*; software used to prepare material for publication: *SHELXTL* and *PLATON* (Spek, 2009 ▶).

## Supplementary Material

Crystal structure: contains datablocks global, I. DOI: 10.1107/S1600536810043230/hb5695sup1.cif

Structure factors: contains datablocks I. DOI: 10.1107/S1600536810043230/hb5695Isup2.hkl

Additional supplementary materials:  crystallographic information; 3D view; checkCIF report

## Figures and Tables

**Table 1:** Hydrogen-bond geometry (Å, °) *Cg*3 is the centroid of the C17–C22 ring.

*D*—H⋯*A*	*D*—H	H⋯*A*	*D*⋯*A*	*D*—H⋯*A*
O1*W*—H1*W*1⋯O3^i^	0.88 (4)	1.97 (4)	2.831 (3)	164 (3)
O1*W*—H2*W*1⋯O1^ii^	0.92 (5)	2.04 (5)	2.944 (3)	167 (4)
C1—H1*A*⋯O1*W*^iii^	0.93	2.24	3.170 (3)	179
C2—H2*A*⋯O1*W*^iv^	0.93	2.44	3.229 (3)	143
C4—H4*A*⋯O1^v^	0.93	2.52	3.406 (2)	160
C6—H6*A*⋯O2	0.93	2.55	3.453 (3)	164
C13—H13*A*⋯O2	0.93	2.51	3.414 (3)	164
C14—H14*A*⋯O2	0.96	2.51	3.106 (3)	120
C14—H14*B*⋯O3^vi^	0.96	2.58	3.393 (3)	143
C9—H9*A*⋯*Cg*3^v^	0.93	2.93	3.650 (2)	135
C12—H12*A*⋯*Cg*3	0.93	2.95	3.760 (2)	147

Symmetry codes: (i) 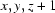; (ii) 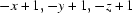; (iii) 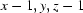; (iv) 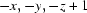; (v) 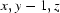; (vi) 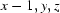.

**Fig. 1.**
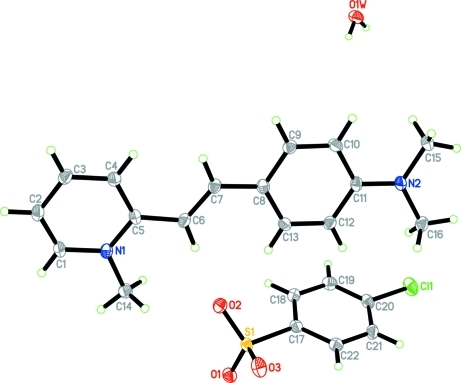
The asymmetric unit of (I) showing 50% probability displacement ellipsoids.

**Fig. 2.**
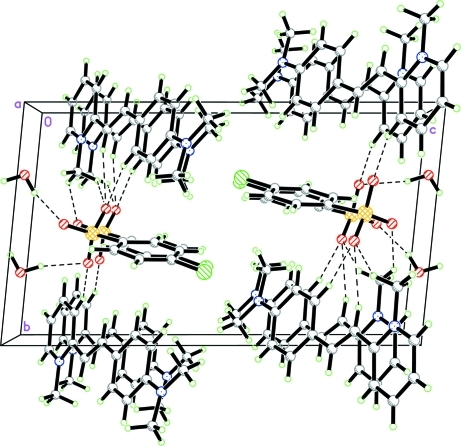
The crystal packing of (I) viewed along the a axis. The O—H···O hydrogen bonds and weak C—H···O interactions are drawn as dashed lines.

## supplementary crystallographic information

### Comment

Our research group have designed and synthesized some quaternary ammonium compounds including pyridinium derivatives. However, there are very few researches in the area of styryl pyridinium dyes being used as antibacterial agents. Based on the knowledge gathered since a very long time ago (Armitage *et al.*, 1929; Browning *et al.*, 1922; Wainwright & Kristiansen, 2003), we found that styryl pyridinium compounds possess high activity against both susceptible and methicillin-resistant *Staphylococcus aureus* (MRSA) (Chanawanno *et al.*, 2010). This interesting anti-MRSA activity of the styryl pyridinium compounds trigger an encouragement to perform further investigation of these compounds in order to act against the powerful superbug MRSA which can overcome commonly used antibacterial drugs (Wainwright, 2008). Our bacterial assay results show that the title compound was moderately active against MRSA with the minimum inhibition concentration (MIC) = 37.5 µg/ml. Herein its crystal structure is reported.

Fig. 1 shows the asymmetric unit of the title compound (I) which consists of the C_16_H_19_N_2_^+^ cation, C_6_H_4_ClO_3_S^-^ anion and one H_2_O molecule. The cation exists in the *E* configuration with respect to the C6═C7 double bond [1.349 (3) Å]. The cation is slightly twisted with the dihedral angle between the C1–C5/N1 pyridinium and the C8–C13 benzene rings being 9.33 (10)° and with the torsion angles C5—C6—C7—C8 = -178.7 (2)°. The two methyl groups of dimethylamino moiety are slightly twisted from the mean plane of the attached C8–C13 ring as indicated by the torsion angles C15—N2—C11—C10 = -2.5 (3)° and C16—N2—C11—C12 = -9.1 (3)°. The cation and anion are inclined to each other which indicated by the dihedral angles between the C17–C22 benzene ring of anion and pyridinium and C8–C13 benzene rings of cation being 79.19 (10) and 70.20 (10)°, respectively. The bond lengths (Allen *et al.*, 1987) and angles in (I) are in normal ranges and comparable with a related structure (Chantrapromma *et al.*, 2010).

In the crystal packing, all O atoms of the sulfonate group are involved in weak C—H···O interactions (Table 1). The cation is linked to both the anion and water molecule by weak C—H···O interactions, and the anion is linked to the water molecule by O—H···O hydrogen bond. These three molecules are linked into chains along the *b* axis (Table 1, Fig. 2). These chains are stacked along the the *a* axis (Fig. 2) by π–π interactions with the distances *Cg*_1_···*Cg*_1_ = 3.6429 (12) Å (symmetry code: -*x*, -*y*, -*z*) and *Cg*_1_···*Cg*_2_ = 3.6879 (12) Å (symmetry code: -1 + *x*, *y*, *z*). C—H···π interactions were also observed (Table 1); *Cg*_1_, *Cg*_2_ and *Cg*_3_ are the centroids of the C1–C5/N1, C8–C13 and C17–C22 rings, respectively.

### Experimental

The title compound was prepared by the reported procedure (Chanawanno *et al.*, 2010). Orange blocks of (I) were recrystallized from methanol by slow evaporation of the solvent at room temperature after a few weeks, m.p. 516–517 K.

### Refinement

Water H atoms were located in difference maps and refined isotropically. The remaining H atoms were placed in calculated positions with d(C—H) = 0.93 Å, *U*_iso_ = 1.2*U*_eq_(C) for aromatic and CH and 0.96 Å, *U*_iso_ = 1.5*U*_eq_(C) for CH_3_ atoms. A rotating group model was used for the methyl groups. The highest residual electron density peak is located at 0.69 Å from C11 and the deepest hole is located at 0.67 Å from S1.

### Crystal data

**Table:**

C_16_H_19_N_2_^+^·C_6_H_4_ClO_3_S^−^·H_2_O	*Z* = 2
*M**_r_* = 448.96	*F*(000) = 472
Triclinic, *P*1	*D*_x_ = 1.397 Mg m^−^^3^
Hall symbol: -P 1	Melting point = 516–517 K
*a* = 6.3895 (1) Å	Mo *K*α radiation, λ = 0.71073 Å
*b* = 9.8739 (2) Å	Cell parameters from 6156 reflections
*c* = 17.0074 (3) Å	θ = 1.2–30.0°
α = 95.721 (1)°	µ = 0.31 mm^−^^1^
β = 90.500 (1)°	*T* = 100 K
γ = 91.260 (1)°	Block, orange
*V* = 1067.32 (3) Å^3^	0.31 × 0.10 × 0.05 mm

### Data collection

**Table:**

Bruker APEX DUO CCD diffractometer	6156 independent reflections
Radiation source: sealed tube	4327 reflections with *I* > 2σ(*I*)
graphite	*R*_int_ = 0.065
φ and ω scans	θ_max_ = 30.0°, θ_min_ = 1.2°
Absorption correction: multi-scan (*SADABS*; Bruker, 2009)	*h* = −8→8
*T*_min_ = 0.912, *T*_max_ = 0.984	*k* = −12→13
23113 measured reflections	*l* = −23→23

### Refinement

**Table:**

Refinement on *F*^2^	Primary atom site location: structure-invariant direct methods
Least-squares matrix: full	Secondary atom site location: difference Fourier map
*R*[*F*^2^ > 2σ(*F*^2^)] = 0.057	Hydrogen site location: inferred from neighbouring sites
*wR*(*F*^2^) = 0.135	H atoms treated by a mixture of independent and constrained refinement
*S* = 1.03	*w* = 1/[σ^2^(*F*_o_^2^) + (0.0548*P*)^2^ + 0.637*P*] where *P* = (*F*_o_^2^ + 2*F*_c_^2^)/3
6156 reflections	(Δ/σ)_max_ = 0.001
282 parameters	Δρ_max_ = 0.47 e Å^−^^3^
0 restraints	Δρ_min_ = −0.43 e Å^−^^3^

### Special details

**Table:**

Experimental. The crystal was placed in the cold stream of an Oxford Cryosystems Cobra open-flow nitrogen cryostat (Cosier & Glazer, 1986) operating at 100.0 (1) K.
Geometry. All e.s.d.'s (except the e.s.d. in the dihedral angle between two l.s. planes) are estimated using the full covariance matrix. The cell e.s.d.'s are taken into account individually in the estimation of e.s.d.'s in distances, angles and torsion angles; correlations between e.s.d.'s in cell parameters are only used when they are defined by crystal symmetry. An approximate (isotropic) treatment of cell e.s.d.'s is used for estimating e.s.d.'s involving l.s. planes.
Refinement. Refinement of *F*^2^ against ALL reflections. The weighted *R*-factor *wR* and goodness of fit *S* are based on *F*^2^, conventional *R*-factors *R* are based on *F*, with *F* set to zero for negative *F*^2^. The threshold expression of *F*^2^ > σ(*F*^2^) is used only for calculating *R*-factors(gt) *etc*. and is not relevant to the choice of reflections for refinement. *R*-factors based on *F*^2^ are statistically about twice as large as those based on *F*, and *R*- factors based on ALL data will be even larger.

### Fractional atomic coordinates and isotropic or equivalent isotropic displacement parameters (Å^2^)

**Table:**

	*x*	*y*	*z*	*U*_iso_*/*U*_eq_	
N1	0.0246 (3)	0.11936 (19)	0.10897 (11)	0.0156 (4)	
N2	1.2149 (3)	0.17645 (19)	0.39494 (11)	0.0193 (4)	
C1	−0.1576 (3)	0.0753 (2)	0.07232 (13)	0.0173 (4)	
H1A	−0.2417	0.1374	0.0500	0.021*	
C2	−0.2199 (3)	−0.0591 (2)	0.06768 (13)	0.0188 (5)	
H2A	−0.3458	−0.0881	0.0431	0.023*	
C3	−0.0922 (3)	−0.1511 (2)	0.10026 (13)	0.0189 (5)	
H3A	−0.1307	−0.2429	0.0969	0.023*	
C4	0.0920 (3)	−0.1056 (2)	0.13766 (13)	0.0174 (4)	
H4A	0.1774	−0.1676	0.1594	0.021*	
C5	0.1533 (3)	0.0327 (2)	0.14361 (12)	0.0157 (4)	
C6	0.3439 (3)	0.0866 (2)	0.18323 (13)	0.0167 (4)	
H6A	0.3830	0.1767	0.1785	0.020*	
C7	0.4671 (3)	0.0127 (2)	0.22659 (13)	0.0167 (4)	
H7A	0.4256	−0.0776	0.2294	0.020*	
C8	0.6572 (3)	0.0584 (2)	0.26937 (13)	0.0162 (4)	
C9	0.7629 (3)	−0.0320 (2)	0.31341 (13)	0.0173 (4)	
H9A	0.7088	−0.1201	0.3143	0.021*	
C10	0.9449 (3)	0.0052 (2)	0.35563 (13)	0.0181 (4)	
H10A	1.0101	−0.0573	0.3845	0.022*	
C11	1.0323 (3)	0.1382 (2)	0.35494 (13)	0.0161 (4)	
C12	0.9253 (3)	0.2302 (2)	0.31130 (13)	0.0168 (4)	
H12A	0.9784	0.3185	0.3103	0.020*	
C13	0.7425 (3)	0.1910 (2)	0.26998 (13)	0.0162 (4)	
H13A	0.6747	0.2537	0.2420	0.019*	
C14	0.0814 (4)	0.2658 (2)	0.10930 (14)	0.0198 (5)	
H14A	0.1017	0.3053	0.1628	0.030*	
H14B	−0.0291	0.3115	0.0849	0.030*	
H14C	0.2084	0.2754	0.0804	0.030*	
C15	1.3197 (4)	0.0833 (3)	0.44178 (15)	0.0237 (5)	
H15A	1.2260	0.0554	0.4813	0.036*	
H15B	1.4412	0.1278	0.4670	0.036*	
H15C	1.3614	0.0048	0.4082	0.036*	
C16	1.3169 (3)	0.3058 (2)	0.38333 (14)	0.0204 (5)	
H16A	1.3290	0.3140	0.3278	0.031*	
H16B	1.4538	0.3098	0.4072	0.031*	
H16C	1.2352	0.3789	0.4073	0.031*	
Cl1	1.18810 (10)	0.69360 (7)	0.46691 (4)	0.02879 (16)	
S1	0.57475 (8)	0.54114 (6)	0.17948 (3)	0.01647 (13)	
O1	0.4692 (2)	0.66863 (16)	0.17231 (10)	0.0204 (3)	
O2	0.4357 (3)	0.43430 (17)	0.20239 (10)	0.0249 (4)	
O3	0.7023 (3)	0.50131 (18)	0.11086 (10)	0.0247 (4)	
C17	0.7541 (3)	0.5767 (2)	0.26013 (13)	0.0164 (4)	
C18	0.6803 (3)	0.5782 (2)	0.33656 (13)	0.0192 (5)	
H18A	0.5409	0.5554	0.3451	0.023*	
C19	0.8147 (4)	0.6137 (2)	0.40055 (14)	0.0209 (5)	
H19A	0.7663	0.6153	0.4520	0.025*	
C20	1.0214 (3)	0.6468 (2)	0.38611 (13)	0.0190 (5)	
C21	1.0976 (3)	0.6445 (2)	0.31068 (14)	0.0204 (5)	
H21A	1.2371	0.6672	0.3024	0.024*	
C22	0.9634 (3)	0.6076 (2)	0.24660 (14)	0.0184 (4)	
H22A	1.0134	0.6037	0.1953	0.022*	
O1W	0.5623 (3)	0.29024 (19)	0.99640 (12)	0.0270 (4)	
H1W1	0.595 (5)	0.366 (4)	1.026 (2)	0.054 (10)*	
H2W1	0.552 (6)	0.317 (4)	0.946 (3)	0.075 (13)*	

### Atomic displacement parameters (Å^2^)

**Table:**

	*U*^11^	*U*^22^	*U*^33^	*U*^12^	*U*^13^	*U*^23^
N1	0.0147 (8)	0.0158 (9)	0.0156 (9)	−0.0006 (7)	−0.0013 (7)	−0.0013 (7)
N2	0.0156 (9)	0.0188 (10)	0.0236 (10)	−0.0026 (7)	−0.0070 (7)	0.0046 (8)
C1	0.0133 (9)	0.0227 (11)	0.0157 (10)	0.0012 (8)	−0.0015 (8)	0.0004 (9)
C2	0.0155 (10)	0.0239 (12)	0.0164 (11)	−0.0026 (9)	−0.0017 (8)	−0.0008 (9)
C3	0.0196 (10)	0.0178 (11)	0.0188 (11)	−0.0048 (9)	−0.0009 (8)	0.0002 (9)
C4	0.0171 (10)	0.0168 (11)	0.0183 (11)	0.0001 (8)	−0.0018 (8)	0.0018 (8)
C5	0.0132 (9)	0.0197 (11)	0.0139 (10)	0.0018 (8)	−0.0010 (8)	−0.0002 (8)
C6	0.0150 (10)	0.0157 (10)	0.0189 (11)	−0.0024 (8)	−0.0010 (8)	0.0004 (8)
C7	0.0157 (10)	0.0138 (10)	0.0198 (11)	−0.0014 (8)	−0.0010 (8)	−0.0014 (8)
C8	0.0146 (10)	0.0174 (11)	0.0161 (10)	0.0016 (8)	−0.0005 (8)	−0.0006 (8)
C9	0.0181 (10)	0.0134 (10)	0.0200 (11)	−0.0015 (8)	−0.0005 (8)	0.0000 (8)
C10	0.0200 (10)	0.0162 (11)	0.0185 (11)	0.0022 (8)	−0.0028 (8)	0.0040 (8)
C11	0.0137 (9)	0.0202 (11)	0.0139 (10)	−0.0008 (8)	−0.0008 (8)	−0.0010 (8)
C12	0.0164 (10)	0.0167 (11)	0.0171 (10)	−0.0023 (8)	−0.0007 (8)	0.0012 (8)
C13	0.0162 (10)	0.0173 (11)	0.0153 (10)	0.0013 (8)	−0.0004 (8)	0.0025 (8)
C14	0.0194 (10)	0.0152 (11)	0.0249 (12)	−0.0007 (8)	−0.0031 (9)	0.0024 (9)
C15	0.0183 (11)	0.0275 (13)	0.0260 (12)	−0.0007 (9)	−0.0068 (9)	0.0073 (10)
C16	0.0182 (10)	0.0220 (11)	0.0205 (11)	−0.0047 (9)	−0.0029 (8)	0.0005 (9)
Cl1	0.0305 (3)	0.0304 (3)	0.0238 (3)	−0.0009 (3)	−0.0125 (2)	−0.0040 (2)
S1	0.0171 (3)	0.0136 (3)	0.0182 (3)	−0.00053 (19)	−0.0044 (2)	−0.0003 (2)
O1	0.0215 (8)	0.0158 (8)	0.0239 (9)	0.0022 (6)	−0.0041 (6)	0.0017 (6)
O2	0.0251 (8)	0.0192 (8)	0.0306 (10)	−0.0073 (7)	−0.0091 (7)	0.0056 (7)
O3	0.0225 (8)	0.0312 (10)	0.0187 (8)	0.0052 (7)	−0.0026 (6)	−0.0070 (7)
C17	0.0186 (10)	0.0104 (10)	0.0199 (11)	0.0008 (8)	−0.0036 (8)	0.0004 (8)
C18	0.0173 (10)	0.0202 (11)	0.0202 (11)	−0.0008 (9)	0.0001 (8)	0.0023 (9)
C19	0.0257 (11)	0.0207 (11)	0.0159 (11)	0.0005 (9)	0.0002 (9)	0.0001 (9)
C20	0.0212 (11)	0.0161 (11)	0.0191 (11)	0.0019 (9)	−0.0061 (9)	−0.0014 (9)
C21	0.0159 (10)	0.0183 (11)	0.0266 (12)	−0.0019 (9)	−0.0048 (9)	0.0013 (9)
C22	0.0207 (11)	0.0166 (11)	0.0180 (11)	0.0011 (9)	−0.0005 (8)	0.0013 (9)
O1W	0.0358 (10)	0.0209 (9)	0.0238 (10)	−0.0001 (8)	−0.0092 (8)	0.0010 (8)

### Geometric parameters (Å, °)

**Table:**

N1—C1	1.358 (3)	C13—H13A	0.9300
N1—C5	1.371 (3)	C14—H14A	0.9600
N1—C14	1.482 (3)	C14—H14B	0.9600
N2—C11	1.372 (2)	C14—H14C	0.9600
N2—C15	1.447 (3)	C15—H15A	0.9600
N2—C16	1.452 (3)	C15—H15B	0.9600
C1—C2	1.372 (3)	C15—H15C	0.9600
C1—H1A	0.9300	C16—H16A	0.9600
C2—C3	1.387 (3)	C16—H16B	0.9600
C2—H2A	0.9300	C16—H16C	0.9600
C3—C4	1.378 (3)	Cl1—C20	1.750 (2)
C3—H3A	0.9300	S1—O2	1.4495 (17)
C4—C5	1.406 (3)	S1—O3	1.4562 (18)
C4—H4A	0.9300	S1—O1	1.4561 (16)
C5—C6	1.451 (3)	S1—C17	1.782 (2)
C6—C7	1.349 (3)	C17—C18	1.386 (3)
C6—H6A	0.9300	C17—C22	1.391 (3)
C7—C8	1.451 (3)	C18—C19	1.392 (3)
C7—H7A	0.9300	C18—H18A	0.9300
C8—C9	1.402 (3)	C19—C20	1.383 (3)
C8—C13	1.406 (3)	C19—H19A	0.9300
C9—C10	1.385 (3)	C20—C21	1.374 (3)
C9—H9A	0.9300	C21—C22	1.395 (3)
C10—C11	1.417 (3)	C21—H21A	0.9300
C10—H10A	0.9300	C22—H22A	0.9300
C11—C12	1.413 (3)	O1W—H1W1	0.88 (4)
C12—C13	1.386 (3)	O1W—H2W1	0.91 (4)
C12—H12A	0.9300		
			
C1—N1—C5	121.90 (19)	C8—C13—H13A	119.2
C1—N1—C14	117.25 (18)	N1—C14—H14A	109.5
C5—N1—C14	120.85 (17)	N1—C14—H14B	109.5
C11—N2—C15	120.73 (19)	H14A—C14—H14B	109.5
C11—N2—C16	119.76 (18)	N1—C14—H14C	109.5
C15—N2—C16	119.16 (17)	H14A—C14—H14C	109.5
N1—C1—C2	121.1 (2)	H14B—C14—H14C	109.5
N1—C1—H1A	119.4	N2—C15—H15A	109.5
C2—C1—H1A	119.4	N2—C15—H15B	109.5
C1—C2—C3	118.99 (19)	H15A—C15—H15B	109.5
C1—C2—H2A	120.5	N2—C15—H15C	109.5
C3—C2—H2A	120.5	H15A—C15—H15C	109.5
C4—C3—C2	119.6 (2)	H15B—C15—H15C	109.5
C4—C3—H3A	120.2	N2—C16—H16A	109.5
C2—C3—H3A	120.2	N2—C16—H16B	109.5
C3—C4—C5	121.2 (2)	H16A—C16—H16B	109.5
C3—C4—H4A	119.4	N2—C16—H16C	109.5
C5—C4—H4A	119.4	H16A—C16—H16C	109.5
N1—C5—C4	117.15 (18)	H16B—C16—H16C	109.5
N1—C5—C6	119.21 (19)	O2—S1—O3	114.41 (11)
C4—C5—C6	123.64 (19)	O2—S1—O1	113.10 (10)
C7—C6—C5	123.3 (2)	O3—S1—O1	112.03 (10)
C7—C6—H6A	118.3	O2—S1—C17	105.34 (10)
C5—C6—H6A	118.3	O3—S1—C17	105.74 (10)
C6—C7—C8	127.2 (2)	O1—S1—C17	105.26 (10)
C6—C7—H7A	116.4	C18—C17—C22	120.5 (2)
C8—C7—H7A	116.4	C18—C17—S1	118.96 (16)
C9—C8—C13	117.25 (18)	C22—C17—S1	120.51 (18)
C9—C8—C7	119.4 (2)	C17—C18—C19	120.0 (2)
C13—C8—C7	123.38 (19)	C17—C18—H18A	120.0
C10—C9—C8	122.3 (2)	C19—C18—H18A	120.0
C10—C9—H9A	118.9	C20—C19—C18	118.8 (2)
C8—C9—H9A	118.9	C20—C19—H19A	120.6
C9—C10—C11	120.26 (19)	C18—C19—H19A	120.6
C9—C10—H10A	119.9	C21—C20—C19	121.9 (2)
C11—C10—H10A	119.9	C21—C20—Cl1	119.68 (17)
N2—C11—C12	121.0 (2)	C19—C20—Cl1	118.42 (18)
N2—C11—C10	121.27 (19)	C20—C21—C22	119.3 (2)
C12—C11—C10	117.72 (18)	C20—C21—H21A	120.4
C13—C12—C11	121.0 (2)	C22—C21—H21A	120.4
C13—C12—H12A	119.5	C17—C22—C21	119.5 (2)
C11—C12—H12A	119.5	C17—C22—H22A	120.3
C12—C13—C8	121.5 (2)	C21—C22—H22A	120.3
C12—C13—H13A	119.2	H1W1—O1W—H2W1	104 (3)
			
C5—N1—C1—C2	−0.7 (3)	C9—C10—C11—N2	−178.6 (2)
C14—N1—C1—C2	178.5 (2)	C9—C10—C11—C12	1.1 (3)
N1—C1—C2—C3	−0.8 (3)	N2—C11—C12—C13	179.1 (2)
C1—C2—C3—C4	1.1 (3)	C10—C11—C12—C13	−0.7 (3)
C2—C3—C4—C5	0.1 (3)	C11—C12—C13—C8	−0.4 (3)
C1—N1—C5—C4	1.9 (3)	C9—C8—C13—C12	1.0 (3)
C14—N1—C5—C4	−177.3 (2)	C7—C8—C13—C12	−179.6 (2)
C1—N1—C5—C6	−178.8 (2)	O2—S1—C17—C18	−39.6 (2)
C14—N1—C5—C6	2.0 (3)	O3—S1—C17—C18	−161.15 (18)
C3—C4—C5—N1	−1.6 (3)	O1—S1—C17—C18	80.12 (19)
C3—C4—C5—C6	179.1 (2)	O2—S1—C17—C22	142.77 (18)
N1—C5—C6—C7	172.1 (2)	O3—S1—C17—C22	21.3 (2)
C4—C5—C6—C7	−8.6 (4)	O1—S1—C17—C22	−97.47 (19)
C5—C6—C7—C8	−178.7 (2)	C22—C17—C18—C19	1.5 (3)
C6—C7—C8—C9	178.2 (2)	S1—C17—C18—C19	−176.11 (18)
C6—C7—C8—C13	−1.1 (4)	C17—C18—C19—C20	−0.2 (3)
C13—C8—C9—C10	−0.5 (3)	C18—C19—C20—C21	−0.5 (4)
C7—C8—C9—C10	−179.9 (2)	C18—C19—C20—Cl1	179.14 (18)
C8—C9—C10—C11	−0.5 (3)	C19—C20—C21—C22	−0.1 (3)
C15—N2—C11—C12	177.8 (2)	Cl1—C20—C21—C22	−179.73 (17)
C16—N2—C11—C12	−9.1 (3)	C18—C17—C22—C21	−2.1 (3)
C15—N2—C11—C10	−2.5 (3)	S1—C17—C22—C21	175.48 (17)
C16—N2—C11—C10	170.6 (2)	C20—C21—C22—C17	1.4 (3)

### Hydrogen-bond geometry (Å, °)

**Table:**

*D*—H···*A*	*D*—H	H···*A*	*D*···*A*	*D*—H···*A*
O1W—H1W1···O3^i^	0.88 (4)	1.97 (4)	2.831 (3)	164 (3)
O1W—H2W1···O1^ii^	0.92 (5)	2.04 (5)	2.944 (3)	167 (4)
C1—H1A···O1W^iii^	0.93	2.24	3.170 (3)	179
C2—H2A···O1W^iv^	0.93	2.44	3.229 (3)	143
C4—H4A···O1^v^	0.93	2.52	3.406 (2)	160
C6—H6A···O2	0.93	2.55	3.453 (3)	164
C13—H13A···O2	0.93	2.51	3.414 (3)	164
C14—H14A···O2	0.96	2.51	3.106 (3)	120
C14—H14B···O3^vi^	0.96	2.58	3.393 (3)	143
C9—H9A···Cg3^v^	0.93	2.93	3.650 (2)	135
C12—H12A···Cg3	0.93	2.95	3.760 (2)	147

Symmetry codes: (i) *x*, *y*, *z*+1; (ii) −*x*+1, −*y*+1, −*z*+1; (iii) *x*−1, *y*, *z*−1; (iv) −*x*, −*y*, −*z*+1; (v) *x*, *y*−1, *z*;  (vi) *x*−1, *y*, *z*.
